# Artificial Intelligence in Bulk and Single-Cell RNA-Sequencing Data to Foster Precision Oncology

**DOI:** 10.3390/ijms22094563

**Published:** 2021-04-27

**Authors:** Marco Del Giudice, Serena Peirone, Sarah Perrone, Francesca Priante, Fabiola Varese, Elisa Tirtei, Franca Fagioli, Matteo Cereda

**Affiliations:** 1 Cancer Genomics and Bioinformatics Unit, IIGM—Italian Institute for Genomic Medicine, c/o IRCCS, Str. Prov.le 142, km 3.95, 10060 Candiolo, TO, Italy; delgiudice.borsisti@iigm.it (M.D.G.); serena.peirone@edu.unito.it (S.P.); sarah.perrone@edu.unito.it (S.P.); priante.borsisti@iigm.it (F.P.); varese.borsisti@iigm.it (F.V.); 2 Candiolo Cancer Institute, FPO—IRCCS, Str. Prov.le 142, km 3.95, 10060 Candiolo, TO, Italy; 3 Department of Physics and INFN, Università degli Studi di Torino, via P.Giuria 1, 10125 Turin, Italy; 4 Department of Physics, Università degli Studi di Torino, via P.Giuria 1, 10125 Turin, Italy; 5 Department of Life Science and System Biology, Università degli Studi di Torino, via Accademia Albertina 13, 10123 Turin, Italy; 6 Paediatric Onco-Haematology Division, Regina Margherita Children’s Hospital, City of Health and Science of Turin, 10126 Turin, Italy; elisa.tirtei@gmail.com (E.T.); franca.fagioli@unito.it (F.F.); 7 Department of Public Health and Paediatric Sciences, University of Torino, 10124 Turin, Italy

**Keywords:** artificial intelligence, RNA sequencing, cancer heterogeneity

## Abstract

Artificial intelligence, or the discipline of developing computational algorithms able to perform tasks that requires human intelligence, offers the opportunity to improve our idea and delivery of precision medicine. Here, we provide an overview of artificial intelligence approaches for the analysis of large-scale RNA-sequencing datasets in cancer. We present the major solutions to disentangle inter- and intra-tumor heterogeneity of transcriptome profiles for an effective improvement of patient management. We outline the contributions of learning algorithms to the needs of cancer genomics, from identifying rare cancer subtypes to personalizing therapeutic treatments.

## 1. Introduction

Artificial intelligence (AI) is becoming a fundamental asset for healthcare and life science research. Despite being in its infancy, research activities employing AI are changing our understanding and vision of science. The European Commission has recently estimated that 13% of global venture capital investments (i.e., ~5 billion of Euros) are for start-ups dedicated to AI application in medicine [1]. This commitment reflects the interest in the potential of AI to improve healthcare. Precision medicine is a new approach to health. In the last decade, the generation of Big Data through genome sequencing (i.e., genomic Big Data), the collection of clinical data, and the growth of bioinformatics has made it possible to identify the genetic causes responsible for onset and progression of diseases and to support the clinical management of patients. Despite the high expectations, personalized therapeutic treatments still remain limited. A breakdown is the lack of AI infrastructure and models capable of supporting the constant generation of genomic Big Data [2]. Consequently, the challenge remains how to interpret the variety of information contained in these data [3].

The need for AI models is even more evident in complex diseases such as cancer. The heterogeneity that characterizes Big Data is amplified in cancer, where diversity not only manifests itself across individuals (i.e., inter-tumor) but also within each tumor (i.e., intra-tumor) [4]. So far, cancer sequencing projects have made available genomic profiles for thousands of biological samples, corresponding to petabytes of genetic information [5]. With the introduction of single-cell technologies, the complexity of genomic information has grown rapidly. This heterogeneity represents the major hurdle to achieve effective precision oncology. Therefore, AI is the pivotal tool to exploit the information available in genomic Big Data and ultimately “deliver” a medicine of precision. The COVID-19 pandemic has opened up new possibilities for AI development. The pandemic has increased the use of AI in biomedical research: from remotely monitoring patients, to predicting the spread of the SARS-CoV-2 coronavirus or in developing new drugs [6,7]. The pandemic has also brought about new clinical practices, primarily the use of mRNA vaccines. This technological leap forward gives the possibility of accelerating the delivery of similar therapies to cancer [8].

Transcriptomics generally refers to the high-throughput profiling of all RNA species produced by cells. Among genomic Big Data, transcriptomics has seen an explosive growth in recent years [9]. RNA sequencing (RNA-seq) profiles dynamic biological processes that are active in a population of cells or in single cells. Assessing the complexity of these profiles could inform the discovery of new biomarkers and therapeutic targets. Since RNA-seq screenings are becoming part of precision medicine trials [10,11], AI mining of these data is thus required to determine novel clinical targets.

In this paper, we provide an overview of AI approaches applied to high-volume bulk and single-cell RNA-seq in cancer genomics and precision oncology. We do not intend to provide a comprehensive characterization of all published AI methods and their technical details. By contrast, we illustrate the major AI solutions to disentangle the heterogeneity of cancer transcriptomes for an effective improvement of patient management. We explain distinct strategies to face the “heterogeneity challenge”. We then outline some of the major contributions of applying AI to the needs of cancer genomics, from identifying rare cancer subtypes to personalizing treatment for individuals.

## 2. AI in the Era of Transcriptomic Big Data

**Figure 1:**
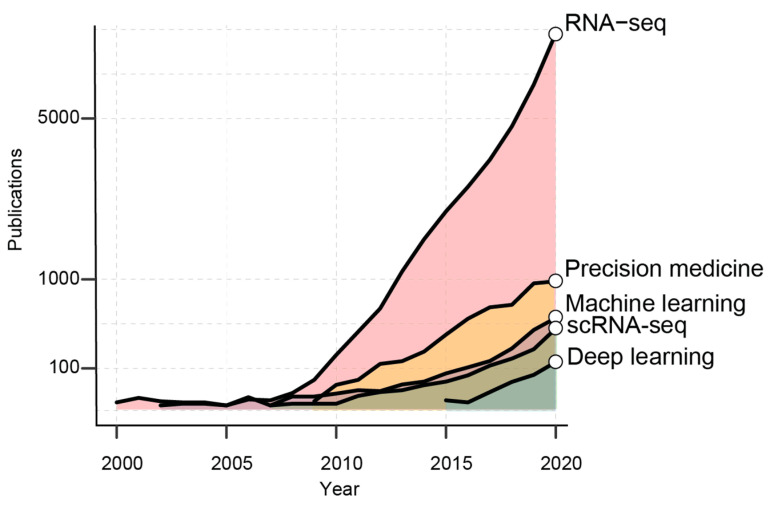
The graph shows the number of PubMed publications per years containing the reported keywords.

From the first drafts of the human genome [12], 20 years ago, the number of scientific works employing sequencing data has exponentially increased (Figure 1). RNA-seq has become a widespread tool to profile cancer transcriptome at both population and single-cell level.

So far, genomic screenings have made available more than 106,585 RNA-seq samples (Table 1) and this number is constantly increasing.

**Figure 2:**
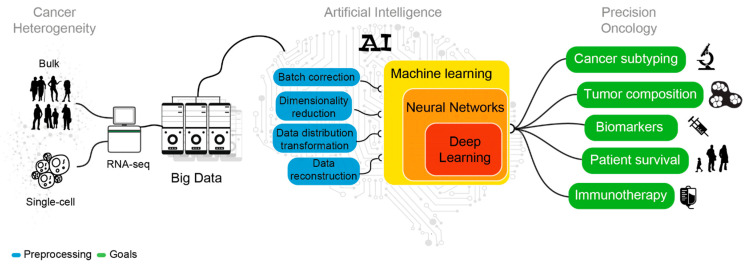
Sketch representing the analyses needed to decipher cancer heterogeneity and achieve an effective precision oncology.

The availability of these data seizes the opportunity to boost the development of novel diagnostic tools and targeted treatments. Indeed, the implementation of AI models has increased in the last 10 years, as machine-learning [13] (ML) and, recently, as deep-learning [14] methods (DL, Figure 1). These learning methods effectively leverage the variability of Big Data to achieve consistent predictions without the need of modeling the system of interest [15]. In the flavor of supervised, semi-supervised and unsupervised, AI algorithms can be employed to capture dependencies, make predictions and recognize patterns in heterogeneous datasets [13]. AI approaches are commonly used to solve regression, classification, dimensionality reduction and clustering tasks. Being part of AI, ML and DL aim at performing tasks that normally require human intelligence. ML and DL accomplish similar tasks with distinct mathematical approaches. While ML algorithms still need human guidance to improve their predictions, DL methods can autonomously determine the accuracy of a prediction. Overall, DL is part of ML where algorithms are generally based on artificial neural networks (NNs), the closest representation of the human brain [16] (Figure 2).

In cancer transcriptomics, ML and DL models have been applied to classify different cancer subtypes and cell populations [17,18,19,20], characterize tumor immune microenvironment [21,22,23,24,25], discover new prognostic biomarkers [26,27,28], assess and predict disease recurrence and patient survival [29,30,31,32], identify new putative actionable vulnerabilities [33,34], and predict tumor antigen immunogenicity [35] (Figure 2).

Disentangling Big Data heterogeneity is the major challenge that researchers have to face to gain novel scientific insights. When learning from real transcriptomic data, the heterogeneity increases due to the variable expression of genes across samples driven by genetic, environmental, demographic, and technical factors [36]. In cancer, the complexity is additionally hampered by the intra- and inter-tumoral heterogeneity of samples [37]. Nevertheless, the constant sequencing of transcriptomes, and thus the increasing volume of data, represents on its own a solid ground for the application of learning approaches to disentangle the noise from the true biological signal.

However, for an effective application of AI algorithms to cancer transcriptomics, the availability of highly-curated datasets is fundamental [38]. Defining an appropriate training dataset with well-defined features is the first important step to ensure better performance of AI models, particularly when integrating data from different sources [38,39]. In this view, data repositories (e.g., refine.bio, RNAseqDB) have been developed to uniformly process and normalize cancer transcriptomes from publicly available sources [40,41]. The use of harmonized and standardized data becomes crucial for the successful translation of AI predictions to the clinical practice.

Finally, to boost the application of AI in precision oncology, it is of primary importance to provide the scientific community with the code and data behind the AI model. This practice is fundamental to ensure the reproducibility and transparency of results [42]. Nowadays, researchers have the opportunity to favor method shareability by implementing them in popular machine learning frameworks, such as PyTorch [43] and Keras [44], and uploading the trained models on dedicated repositories like Kipoi [45].

## 3. Managing the Heterogeneity of Cancer Transcriptomes

**Figure 3:**
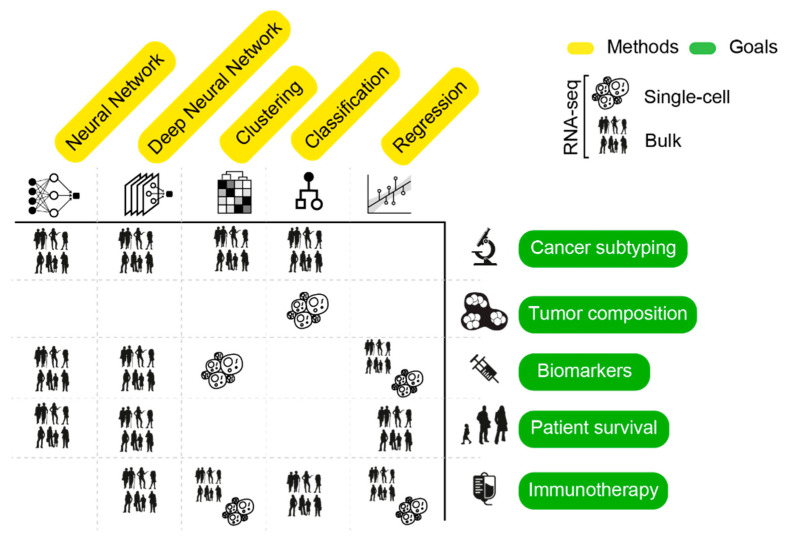
Graphical summary of AI approaches (columns) applied to solve tasks (rows) presented in this review. Cells show the RNA-seq data type used for the analysis. The “immunotherapy” task includes assessment of tumor microenvironment and identification of neoepitopes.

Heterogeneity of cancer transcriptomes can arise from both technical and biological confounders [36]. Several strategies have been developed to increase signal-to-noise ratio in large-scale RNA-seq datasets. In this view, a crucial step for the effectiveness of AI algorithms is data preprocessing [46]. To remove the effect of confounders that can lead to false data dependencies, techniques such as batch-correction, dimensionality reduction, data discretization and feature selection are normally employed. These strategies can be used independently or in combination, either as a core or a result of AI. Below, we explore the tight link between these approaches and learning strategies. All methods that we report are listed in Table 2 and summarized in Figure 3.

### 3.1. Batch-Correction of Technical Heterogeneity

Technical heterogeneity rises during the experimental generation of sequencing data. In particular, cancer transcriptomes can be profiled (i) from distinct sample types; (ii) using different protocols and platforms; and (iii) processed in unconnected laboratories by specific users in separate times. This “batch-specific” heterogeneity confounds the real biological signal of large-scale datasets [79]. Therefore, an effective removal of batch-effects is an essential step during data integration. Conventionally, batch-effects in bulk RNA-seq are resolved using ML regression models [80]. With the advent of single-cell RNA-seq, different non-linear, transfer-learning, supervised and unsupervised DL approaches have been successfully proposed. For instance, NNs trained to minimize discrepancies between distributions of replicates have been shown to attenuate technical confounders [47]. Autoencoders, or unsupervised NNs, that gradually remove batch-effect over iterations have shown to amplify biological signals by transferring information across batches [48]. Similarly, unsupervised deep embedding NNs that simultaneously learn gene expression representations and cluster assignments demonstrated a great removal of batch-effects while preserving biological heterogeneity [49]. Supervised mutual nearest neighbor detection within cell types revealed an improved clustering of cell types across batches [50]. Overall, DL approaches showed the best reduction of batch-effects can be achieved while learning from clustering data across iterations.

### 3.2. Dimensionality Reduction Approaches

Heterogeneity emerges from the high dimensionality of transcriptomic datasets, which profile thousands of RNA isoforms. These profiles result in lists of fixed-length vectors of real values, namely features, which are highly variable in a specific range. The extremely high number of heterogeneous features prevents direct identification of biological similarities across samples under a phenotype of interest. In this view, dimensionality reduction is a useful pre-processing approach to remove confounders, speed up learning methods and improve their accuracy in detecting similarities [81]. These techniques perform dimensionality reduction by either extracting novel features or selecting the best informative features from the original dataset.

#### 3.2.1. Feature Extraction

Principal Component Analysis (PCA) is conventionally used for dimensionality reduction and exploratory analyses of transcriptomic profiles [82]. PCA aims at extracting a limited number of new features that maximize the variability present in the original data through a linear approach. In precision oncology, different PCA-based approaches showed to enhance the consistency of cancer subtyping [83], the discovery of putative novel therapeutic targets [84], and the identification of prognostic gene signatures [85].

Being a linear approach, the accuracy of PCA is limited when dealing with large-scale RNA-seq datasets [86]. To overcome this issue, non-linear methods, such as T-distributed Stochastic Neighbor Embedding (t-SNE) and Uniform Manifold Approximation and Projection (UMAP), have been recently employed to capture variability of cancer transcriptomes. t-SNE and UMAP aim at deconvoluting relationships between neighbors in high-volume datasets [87,88], with different implementations [89,90]. These unsupervised non-linear dimensionality reduction approaches showed to be effective in separating cell types in scRNA-seq datasets. Recently, these methods have also been shown to capture the heterogeneity of large-scale bulk RNA-seq [91]. Applied to thousands of cancer transcriptomes, t-SNE revealed small gene signatures correlating with long-term survival in the majority of tumor types [92].

Feature extraction can also be performed employing DL approaches. For instance, convolutional NNs can automatically reduce data dimensionality and perform classification tasks. Convolutional NN methods have been successfully implemented in digital pathology. Interestingly, these algorithms have been capable of inferring cancer transcriptomic profiles from histological images [93]. Applied to bulk RNA-seq datasets, CNN revealed a high accuracy to classify cancer subtypes [51] and predict cancer progression [52]. Similarly, DL methods have been designed to improve our understanding of single-cell heterogeneity. Deep generative models, which combine probabilistic models and NNs, have recently been shown to enhance dimensionality reduction of scRNA-seq datasets by preserving their global structure, thus improving the interpretation of results. Applied to a large-scale melanoma dataset, this DL method accurately discriminated tumor cells from microenvironment components [53].

#### 3.2.2. Feature Selection

Feature selection (FS) aims at identifying the most important attributes from heterogeneous high-dimensional data [13]. The choice of an appropriate FS method is fundamental for the identification of the real biological information, especially in precision oncology when searching prognostic gene signature, biomarkers and actionable targets. In terms of AI, the use of a list of selected features reduces overfitting effects and increases model stability, and thus prediction accuracy. It has been shown that NN approaches improve their accuracy when coupled with FS methods [94]. Similarly, regression models based on FS resulted in an improved classification of breast cancer subtypes from bulk RNA-seq datasets [17]. A review of the main feature selection approaches for bulk and single cell transcriptomic data has been recently presented [95].

Despite being pivotal for increasing the accuracy of AI predictions, the presence of extensive correlations between variables in large-scale datasets could reduce the stability of selected features [96]. To mitigate this issue, DL-based feature selection algorithms have been introduced. For instance, NNs have been successfully applied to identify small gene signatures as oncogenic biomarkers from a large-scale pan-cancer RNA-seq dataset [54]. Similarly, the use of polynomial and radial kernels that are pivotal for ML algorithms has been shown to achieve higher accuracy than conventional FS approaches in selecting oncogenic gene signatures from bulk RNA-seq data [55].

### 3.3. Data Distribution Transformation

Since AI methods learn from inputs to predict outputs, the different scale and distribution of features in the training data can impact on model performance, particularly if the algorithm is based on distance measures [97]. For instance, features with a considerable spread of values may result in large error gradients causing NN instability [98]. This holds particularly true for cancer transcriptomes where distinct genes whose expression fluctuates in a small interval can drive the phenotype rather than single genes with large expression spread [99]. Feature centering and scaling is a critical preprocessing step to assure that all variables proportionally contribute to the AI model. These steps are widely used to normalize bulk and single-cell RNA-seq data [100,101]. Feature scaling helps to detect informative gene signature and altered processes from expression data. For instance, rank-based preprocessing normalization of gene expression profiles from bulk RNA-seq experiments has been shown to be effective in determining the real altered pathways in KRAS-driven cancers [56].

Similarly, data discretization is a preprocessing step through which values are divided in a finite number of classes. AI methods, such as classification and clustering, can improve in learning speed and accuracy by discretizing the distribution of numerical input values [102,103]. In this view, we recently demonstrated how discretization of expression profiles boosts the prediction of altered biological processes in large-scale transcriptomic datasets [57]. This preprocessing step allowed us to identify the role of the tumor-suppressor gene, PTEN, in modulating immune-related processes and determine the maximum expression level for which PTEN leads to a worse patient survival. Discretization of isoform-level gene expression profiles has been successfully applied to increase accuracy of glioblastoma subtype classification [58]. Overall, data discretization increases signal-to-noise ratio at the cost of a partial loss of information, which is mitigated by the large quantity of data. Applied to transcriptomic Big Data, this technique offers the chance to extract relevant information while accounting for their intrinsic heterogeneity. However, due to information loss, the choice of an appropriate discretization strategy impacts on the design and performance of the AI model, therefore remaining a non-trivial task [104].

### 3.4. Data Reconstruction: The Sparsity Issue

Features that have many zero values are commonly referred to as sparse, and their presence can lead to overfitting and reduced performances in AI models. The presence of sparse features characterizes single-cell RNA-seq datasets due to experimental limitations [105,106]. Data reconstruction aims at transforming incomplete input values into a corresponding complete set [107]. Several reconstruction methods have been developed to overcome this technical heterogeneity of single-cell transcriptomic profiles. Most of them are autoencoder-based DL algorithms, which use probabilistic data generative processes to reconstruct the observed profiles from low-dimensional or latent space representations [59,60,61,105]. The use of these data reconstruction tools has been demonstrated to enhance performance in recovering biologically meaningful states, improving data clustering and, consequently, differential expression analysis.

## 4. AI Mining of Cancer Transcriptomes

Cancer manifests its genetic heterogeneity with the presence of distinct histological subtypes and tumor microenvironment (TME) compositions across tumors and within the same disease. Inter- and intra-tumor heterogeneity have different clinical implications, making their accurate identification pivotal for therapeutic decisions [37]. As previously mentioned, AI models applied to transcriptomic Big Data have increased the accuracy of cancer classification, biomarker discovery, disease recurrence and patient survival forecast, and understanding of immune regulation.

### 4.1. Assessing Inter-Tumor Heterogeneity: Classification of Cancer Subtypes

One of the most used AI approaches to assess inter-tumor heterogeneity and improve the identification of distinct molecular subtypes using transcriptomic data is unsupervised learning clustering. This technique aims at identifying groups of samples with similar biological features by partitioning data according to similarity measures [108]. Different studies have shown the utility of clustering approaches in detecting molecular features (i.e., gene expression signature) responsible for patient prognosis and management. For example, non-negative matrix factorization clustering of gene expression data has been successfully exploited to improve ovarian cancer subtyping [62]. This approach identified distinct molecular subtypes associated with different patient survival and residual disease. Recently, topic modeling has been proposed to enhance the detection of more subtle inter-tumor heterogeneity. Developed for natural language processing, this probabilistic clustering algorithm aims at discovering the hidden “topics” that reflect the biological heterogeneity and enhancing its comprehensive interpretation [109]. Applied to breast and lung cancer RNA-seq datasets, topic modeling outperformed standard clustering algorithms in identifying subtype-specific molecular features and their corresponding clinical outcomes [20].

The use of prior information can be a useful solution to train AI models more effectively [13]. For this reason, when prior knowledge about subtype features is available, supervised methods can be exploited for a more accurate cancer subtyping [17,18,63,65,66]. For instance, feature selection of differentially expressed genes and scoring of system-level properties (e.g., protein-protein interaction network centrality, gene essentiality, gene evolutionary origin, pathway information) has been employed to select gene signatures to train support vector machine (SVM) predictors of cancer recurrence and prognosis [110,111,112,113]. Similarly, the integration of pathway enrichment scores as input of random forest improved breast cancer classification is relative to single-gene signature-based methods [63]. Recently, partition around medoids clustering of metabolism-related gene set activity scores has been used to identify prostate cancer subtypes associated with patient prognosis and therapy response [64].

However, the heterogeneous composition of large-scale datasets can lead to AI models that are biased toward specific subtypes, thus impacting patient management [65]. A Naïve Bayes classifier based on binary rules that define gene expression dependencies within individual samples has been proposed to improve the identification of patient-specific tumor subtypes [65]. This sample-specific approach revealed an improved identification of breast cancer subtypes, regardless of the biological (i.e., tumor cellularity) and technical (i.e., sequencing technology) heterogeneity in the dataset. Similarly, single-sample feature selection of gene signatures combined with multiclass logistic regression achieved the best performance to classify breast cancer subtypes on 4731 RNA-seq expression profiles [17].

DL approaches have shown advantages over supervised ML methods for their ability of automatically extracting features from input data [114]. An autoencoder-based DL approach combining supervised classification and unsupervised clustering revealed the presence of novel breast and bladder cancer subtypes associated with different prognosis [66]. Convolutional NNs have been employed to infer tumor’s primary tissue of origin of metastasis and to guide management of patients with cancer of unknown primary [67]. Again, integrating biological information (i.e., gene set enrichment analysis) in NNs resulted in an improved classification of individual colorectal and breast cancer subtypes relative to canonical ML approaches [18]. A further advantage of embedding prior biological information in AI models is the easier clinical interpretability of the features defining different subtypes, which can foster the development of novel therapeutic strategies.

### 4.2. Deciphering Intra-Tumor Heterogeneity

#### 4.2.1. Defining Cell Types and Clones

Transcriptomic profiling of single cells has allowed direct access to intra-tumor heterogeneity through the identification of cell types and clones composing the tumor mass. Learning clustering represents the commonest approach to identify gene signatures representative of specific cell types [21,22,68,69]. As mentioned above, scRNA-seq data are highly heterogeneous, noisy and sparse. This makes clustering analysis particularly challenging. To face this issue, dimensionality reduction approaches (e.g., principal coordinate analysis (PCoA), t-SNE) are generally employed as preprocessing steps of clustering analysis [21,22,68]. These approaches, followed by a manual revision of the identified gene signatures, have been successfully applied to identify cell types associated with different proliferative states and therapy responses in nasopharyngeal tumors and osteosarcomas [21,22]. Similar to cancer subtyping, the integration of prior knowledge in learning algorithms can be useful to improve the interpretation of intra-tumor heterogeneity. Cell clustering using gene set features derived from enrichment analysis improved glioblastoma subtyping, revealing novel metabolism-associated groups associated with distinct prognostic and therapeutic properties [69]. Similarly, clustering including information about somatic alterations has been shown to improve the accuracy of subclone detection and prediction of subclonal neoantigens in breast cancer and melanoma, respectively [70].

#### 4.2.2. Assessment of TME

The heterogeneous composition of the tumor mass increases the complexity of cancer transcriptomes, making the systematic characterization of TME fundamental for the development of personalized therapies. The fine quantification of tumor-infiltrating immune cells can help to guide the selection and understand the effect of immunotherapeutic approaches. For these reasons, ML methods have been proposed to deconvolute cell-type abundance from bulk transcriptomic profiles of mixed populations. Among others, a ML approach based on non-negative matrix factorization has shown to accurately define cell-type-specific expression signatures exploiting tissue heterogeneity in more than 2300 cancer transcriptomes [24]. In absence of physical cell isolation, this method demonstrated to successfully separate the contribution of malignant cells from immune cells and fibroblasts in both head and neck tumors and melanomas. Similarly, least square regressions have been employed to isolate cell-type-specific contributions while accounting for the presence of uncharacterized cells in melanoma samples [74]. The use of gene sets rather than single genes in a curve-fitting approach has been shown to be effective in defining expression profiles of 60 different cell types from 9947 RNA-seq profiles across 37 cancer types [25].

The application of learning approaches to single-cell transcriptomic profiles of physically isolated cells has improved the characterization of TME composition and interactions. PCA dimensionality reduction followed by joint embedding and clustering approach elucidated the cellular composition of osteosarcoma, showing that TME-based chemotherapy may reduce osteoclast differentiation to osteosarcoma [22]. UMAP-based clustering analysis of scRNA-seq data unveiled the existence of novel subtypes of B cells associated with tumor progression [75].

Finally, the combination of bulk and single-cell transcriptome profiling of tumors can improve the characterization of TME and the selection of personalized therapeutic treatments. For instance, supervised clustering approach of 2269 bulk and 10,434 single-cell colorectal transcriptomes identified a TME-associated chemotherapy resistant gene signature enabling tumor subtyping with potential therapeutic response [76].

Overall, deconvolution AI algorithms represent a powerful tool for improving our understanding of TME composition and the delivery of personalized medicine.

### 4.3. Biomarker Identification

To deliver an effective personalized medicine, the precise identification of patient-specific genetic markers that drive the disease is fundamental. To foster the discovery of novel cancer vulnerabilities, ML and DL have been applied to large-scale transcriptomic profiles and, often, integrated with pharmacogenomics (i.e., drug sensitivity) data. These approaches commonly employ protein-protein interaction network-based feature selection analyses to identify gene signatures associated with drug response, which are then used to train ML classifiers. Recently, an example of these ML frameworks based on ridge regressions has been shown to accurately identify gene signature associated with drug response of colorectal and bladder cancer patients [26]. A similar ML framework employing Cox Proportional Hazards (Cox-PH) regression has been used to determine functional protein-protein interaction subnetworks as prognostic biomarkers in different cancer types [27]. NN classifiers such as restricted Boltzmann machines have been successfully exploited to identify biomarker gene regulatory networks, with available targeting drugs, associated with lung cancer development [71].

The integration of multiple AI techniques can be a handy solution to improve the identification of cancer vulnerabilities. Recently, a combination of three learning algorithms resulted in identifying histone deacetylase inhibitors as potential therapeutic targets for multiple soft tissue sarcomas [33]. In particular, the framework employed (i) NNs to determine gene expression signatures of soft tissue sarcomas relative to healthy tissues, (ii) random forest to identify novel diagnostic markers, and (iii) k-nearest neighbor algorithm to determine prognostic genes.

The analysis of single-cell transcriptomic data can enhance the detection of gene signatures that can discriminate between somatic cells and the other cell types composing the tumor mass. Recently, the combination of dimensionality reduction performed by projected matrix decomposition and clustering through non-negative matrix factorization identified gene signatures of healthy brain cells [28]. Applied to bulk glioblastoma RNA-seq data, these gene signatures successfully predicted patient survival. Similarly, feature selection through maximum relevance minimum redundancy analysis followed by SVM classification revealed glioblastoma-specific biomarkers associated with cancer aggressiveness [72]. As described for bulk RNA-seq, the integration of prior knowledge (e.g., protein-protein, ligand-receptor, regulatory interactions) can also improve biomarker discovery using single-cell transcriptomic profiles. Dimensionality reduction via diffusion map and shared-nearest-neighbor clustering of glioblastoma cells identified potential prognostic biomarkers [73]. Overall, the identification of gene sets as biomarkers rather than single genes provides more comprehensive information on the relevant biological processes responsible for the disease, enlarging the list of novel putative drug targets.

### 4.4. Prediction of Patient Survival

Stratification of patients into groups with different survival probabilities using prognostic biomarkers is pivotal to prioritize treatments and avoid unnecessary therapies [115]. Traditionally, the effect of gene expression on patients’ survival is measured using linear Cox-PH regression models [116]. However, the high-dimensionality of transcriptomic large-scale datasets impacts on the performance of Cox-PH models leading to overfitting issues [30]. For this reason, ML extensions of the Cox-PH model employing random forest have been developed [117]. Recently, NN approaches have been shown to outperform classical survival methods [29,30,31,32]. These algorithms exploit feature selection through NNs to obtain a subset of surrogate prognostic features. The surrogate features are then used in the Cox-PH model to predict the hazard ratios. Applied to transcriptomic data of kidney cancer, surrogate features defined by NNs have been shown to capture real biological processes (i.e., p 53 signaling pathway) responsible for different prognosis of patients [30]. The integration of prior information about drug treatments in NN prognostic models has been shown to improve therapeutic indications according to the predicted effect of treatment options on individual patients [31]. The use of autoencoders in the feature selection step has also been proposed [32].

To address the scarcity of training samples available for specific cancer types, Cox-PH NN generalizations can be exploited using transfer learning approaches [14]. Transfer learning is a ML technique by which a model trained on one setting is exploited on another related setting [118]. Transfer learning has been employed to assess patient survival in pan-cancer RNA-seq datasets [29]. The model showed a higher prognostic performance than competing methods and exploiting risk score backpropagation [119] allowed to assess the biological pathways that impact on patient’s survival outcome in the tested cancer types.

Together, these results show that NN extensions of Cox-PH modeling improve the identification of prognostic gene signature responsible for cancer progression, and thus of putative novel biomarkers.

### 4.5. Identification of Neoepitopes

Neoepitopes are tumor-specific peptides that are presented by antigen-presenting cells through the major histocompatibility complex and recognized by the immune system [35]. Several promising immunotherapeutic anticancer approaches (e.g., vaccines, chimeric antigen receptor and T-cell receptor engineered T cells) rely on the identification of suitable target antigens. However, one of the major obstacles for the broader applicability of such therapies is the lack of targetable tumor-specific antigens for many cancer types [120]. Furthermore, in vitro selection of antigens remains an expensive and difficult task. So far, genomic studies analyzed thousands of sequencing data to identify somatic alterations driving tumor progression, but only somatic mutations have been exploited for their potential to generate novel peptides that can stimulate the immune system. Recent studies have shown that the aberrant alternative splicing that characterizes many cancer types has a stronger potential of generating neoepitopes [121].

NNs have been developed to improve peptide-prediction accuracy and MHC-ligand identification [122] from somatic mutations. To date, these approaches have been proved to be also effective in predicting immunogenic peptides derived from somatic splicing defects in melanoma, B-cell lymphoma and leukemia cell lines, even if lacking clinically relevant validations [123]. An ensemble of ML classifiers (i.e., Naïve Bayes, random forest and SVM) has recently been shown to improve immunogenetic predictions of neoantigens and proposed for ranking their potential effectiveness [77]. Nevertheless, the lack of clinically validated neoantigens on large-scale cohorts limits the efficient training of AI algorithms [124]. To solve this issue, a multimodal recurrent NNs approach integrating mass spectrometry data has been proposed [78]. Overall, the identification of neoantigens from large-scale transcriptomic dataset is still in its infancy and presents considerable opportunities for AI improvements.

## 5. Conclusions

Despite the results achieved so far, the application of AI to cancer transcriptome Big Data for valuable precision oncology is still limited. The complexity of cancer heterogeneity remains the major challenge to disentangle. On the one hand, AI represents the most powerful tool to extract the real biological information from large-scale transcriptomic datasets. As national and international sequencing consortia generate sequencing data, the ability of DL algorithms to capture the hidden relationships responsible for a phenotype without requiring a human supervision will become pivotal for our understanding of diseases and guide personalized therapeutic interventions. On the other hand, AI data mining poses several challenges. Harnessing Big Data carries with it the ‘curse of dimensionality’ phenomenon, or the need of more data when information increases [125,126]. When dimensionality grows, data becomes sparse. Any sample is likely to be more separated from its neighbors at the increase of the space dimensionality. Hence, having data fully representative of the heterogeneity of a phenotype will become more and more complicated as the variables of interest will increase. This holds particularly true for cancer types that are rare and heterogeneous. Dimensionality reduction methods are a solution to mitigate the curse of dimensionality. Similarly, data discretization approaches can help to reduce dimensionality supporting the paradigm of “less is more”. Despite being powerful tools, AI approaches require tailor-made designs to achieve good performances and biologically relevant results. The “black-box” nature of learning algorithms needs to be fully exploited to reach a comprehensive understanding of the cancer phenotype of interest. Improving the interpretability of results of AI models remains an important challenge [127], especially when selecting for therapeutic treatments. However, the integration of prior biological knowledge into the algorithms can guide toward this direction. Combining data from multi omics approaches will provide a deeper understanding of cancer heterogeneity. However, new AI methods will be required to face the resulting curse of dimensionality. Of note, part of cancer transcriptomic data originates from preclinical research employing cell lines and mouse models. Despite the undeniable value of these data, molecular differences between these models and patient tumors call for caution in extending results to the human system [128,129]. Therefore, approaches aiming at delineating the similarities and differences between preclinical and clinical transcriptomes are required for an effective application of AI to improve the patient’s quality of life [130,131].

The demand of AI in precision oncology will go hand in hand with the need of doctors and experts that will be able to translate results into real precision therapeutic decisions and participate actively in the development of learning strategies. In this light, a precision AI-driven oncology will become effectively available on demand.

## Figures and Tables

**Table 1:** The table reports the number of publicly available bulk and single-cell RNA-seq experiments. Data stored in reported repository are frozen at 15 March 2021.

Repository	URL	Bulk	Single-Cell
GDC	portal.gdc.cancer.gov	27,894	18
ENCODE	www.encodeproject.org	2323	7
GEO	www.ncbi.nlm.nih.gov/geo	30,510	2346
SRA	www.ncbi.nlm.nih.gov/sra	1874	6428
St. Jude	www.stjude.cloud	3215	-
ICGC	dcc.icgc.org	12,840	-
GTEx	www.gtexportal.org/home	17,382	-
DepMap	depmap.org/portal	1376	-
Human Cell Atlas	data.humancellatlas.org	-	289
Single Cell Portal	singlecell.broadinstitute.org	-	83

**Table 2:** The table reports all learning approaches reported in the main text with respect to each section.

Section	Method	RNA-Seq Experiment	Authors
Batch-correction of technical heterogeneity	Residual neural network	single-cell	Shaham et al., 2017 [47]
	autoencoder	single-cell	T. Wang et al., 2019 [48]
	Autoencoder and iterative clustering	single-cell	Li et al., 2020 [49]
	Supervised mutual nearest neighbor	single-cell	Yang et al., 2020 [50]
Feature extraction	Convolutional neural network	bulk	Elbashir et al., 2019 [51]
	Convolutional neural network	bulk	López-García et al., 2020 [52]
	Deep generative models	single-cell	Ding et al., 2018 [53]
	Wx, neural network	bulk	Park et al., 2019 [54]
	Double Radial Basis Function Kernels	bulk	Liu et al., 2018 [55]
Data distribution transformation	Rank-based normalization	bulk	Barbie et al., 2009 [56]
	GSECA, Gene Set Enrichment Class Analysis	bulk	Lauria et al., 2020 [57]
	Equal-width, equal-frequency binning, k-means clustering	bulk	Jung et al., 2015 [58]
Data reconstruction: the sparsity issue	AutoImpute, autoencoder	single-cell	Talwar et al., 2018 [59]
	DeepImpute, autoencoder	single-cell	Arisdakessian et al., 2019 [60]
	DCA, autoencoder	single-cell	Eraslan et al., 2019 [61]
Assessing inter-tumor heterogeneity: classification of cancer subtypes	Non-negative matrix factorization	bulk	Wang et al., 2017 [62]
	Topic modeling	bulk	Valle et al., 2020 [20]
	Random forest	bulk	Alcaraz et al., 2017 [63]
	Partition around medoids	bulk	Zhang et al., 2020 [64]
	Naïve Bayes classifier	bulk	Paquet et al., 2015 [65]
	Multiclass logistic regression	bulk	Cascianelli et al., 2020 [17]
	DeepType, neural network	bulk	Chen et al., 2020 [66]
	CUP-AI-Dx, convolutional neural network	bulk	Zhao et al., 2020 [67]
	DeepCC, neural network	bulk	Gao et al., 2019 [18]
Defining cell types and clones	Density clustering	single-cell	Izar et al., 2020 [68]
	Graph-based clustering	single-cell	Chen et al., 2020 [21], Zhou et al., 2020 [22]
	Consensus clustering	single-cell	Garofano et al., 2021 [69]
	DENDRO, kernel-based clustering	single-cell	Zhou et al., 2020 [70]
Biomarker identification	Interaction network and ridge regression	bulk	Kong et al., 2020 [26]
	SIMMS, Interaction network and Cox Proportional Hazards	bulk	Haider et al., 2018 [27]
	ECMarker, Boltzman machines	bulk	Jin et al., 2020 [71]
	Integration of ML techniques	bulk	van IJzendoorn et al., 2019 [33]
	DRjCC, non-negative matrix factorization	single-cell	Wu et al., 2020 [28]
	maximum relevance minimum redundancy, Support vector machine	single-cell	Cheng et al., 2020 [72]
	Diffusion map, shared nearest-neighbor clustering and Cox Proportional Hazards	single-cell	Zhang et al., 2020 [73]
Prediction of patient survival	Cox-nnet, neural network and Cox Proportional Hazards	bulk	Ching et al., 2018 [30]
	DeepSurv, neural network and Cox Proportional Hazards	bulk	Katzman et al., 2018 [31]
	AECOX, autoencoder and Cox Proportional Hazards,	bulk	Huang et al., 2020 [32]
	Neural network and Cox Proportional Hazards	bulk	Qiu et al., 2020 [29]
Assessment of tumor microenvironment	CIBERSORTx, support vector regression	single-cell/bulk	Newman et al., 2015 [24]
	EPIC, least square regression	single-cell/bulk	Racle et al., 2017 [74]
	xCell, non-linear regression	bulk	Aran et al., 2017 [25]
	Graph-based clustering	single-cell	Chen et al., 2020 [75]
	K-means clustering	single-cell/bulk	Zhu et al., 2021 [76]
Identification of neoepitopes	Neopepsee, Naïve Bayes, random forest, support vector machine	bulk	Kim et al., 2018 [77]
	MARIA, multimodal recurrent neural network	bulk	Chen et al., 2019 [78]

## Data Availability

A tutorial to implement AI algorithms in the R scripting language for sample classification and gene signature discovery is available at http://www.ceredalab/AI/index.html and at https://github.com/matteocereda/AI, accessed on 24 April 2021.
